# Next-generation sequencing diagnostics of bacteremia in sepsis (Next GeneSiS-Trial): Study protocol of a prospective, observational, noninterventional, multicenter, clinical trial

**DOI:** 10.1097/MD.0000000000009868

**Published:** 2018-02-09

**Authors:** Thorsten Brenner, Sebastian O. Decker, Silke Grumaz, Philip Stevens, Thomas Bruckner, Thomas Schmoch, Mathias W. Pletz, Hendrik Bracht, Stefan Hofer, Gernot Marx, Markus A. Weigand, Kai Sohn

**Affiliations:** a Department of Anesthesiology, Heidelberg University Hospital, Heidelberg; b Fraunhofer IGB, Stuttgart; c Noscendo GmbH, Duisburg; d Institute of Medical Biometry and Informatics, University of Heidelberg, Heidelberg; e Institute of Infectious Diseases and Infection Control, Jena University Hospital, Jena; f Department of Anaesthesiology, Division of Intensive Care, University Clinic Ulm, Ulm; g Department of Anesthesiology, Westpfalzklinikum, Kaiserslautern; h Department of Intensive Care and Intermediate Care, RWTH University Hospital Aachen, Aachen, Germany.

**Keywords:** bacteremia, blood culture, next generation sequencing, procalcitonin, sepsis, sequential organ failure assessment (SOFA) score

## Abstract

**Background:**

Sepsis remains a major challenge, even in modern intensive care medicine. The identification of the causative pathogen is crucial for an early optimization of the antimicrobial treatment regime. In this context, culture-based diagnostic procedures (e.g., blood cultures) represent the standard of care, although they are associated with relevant limitations. Accordingly, culture-independent molecular diagnostic procedures might be of help for the identification of the causative pathogen in infected patients. The concept of an unbiased sequence analysis of circulating cell-free DNA (cfDNA) from plasma samples of septic patients by next-generation sequencing (NGS) has recently been identified to be a promising diagnostic platform for critically ill patients suffering from bloodstream infections. Although this new approach might be more sensitive and specific than culture-based state-of-the-art technologies, additional clinical trials are needed to exactly define the performance as well as clinical value of a NGS-based approach.

**Methods:**

*Next GeneSiS* is a prospective, observational, noninterventional, multicenter study to assess the diagnostic performance of a NGS-based approach for the detection of relevant infecting organisms in patients with suspected or proven sepsis [according to recent sepsis definitions (sepsis-3)] by the use of the quantitative sepsis indicating quantifier (SIQ) score in comparison to standard (culture-based) microbiological diagnostics. The clinical value of this NGS-based approach will be estimated by a panel of independent clinical specialists, retrospectively identifying potential changes in patients’ management based on NGS results. Further subgroup analyses will focus on the clinical value especially for patients suffering from a failure of empiric treatment within the first 3 days after onset [as assessed by death of the patient or lack of improvement of the patient's clinical condition (in terms of an inadequate decrease of SOFA-score) or persistent high procalcitonin levels].

**Discussion:**

This prospective, observational, noninterventional, multicenter study for the first time investigates the performance as well as the clinical value of a NGS-based approach for the detection of bacteremia in patients with sepsis and may therefore be a pivotal step toward the clinical use of NGS in this indication.

**Trial registration:**

DRKS-ID: DRKS00011911 (registered October 9, 2017) https://www.drks.de/drks_web/navigate.do?navigationId=trial.HTML&TRIAL_ID=DRKS00011911; ClinicalTrials.gov Identifier: NCT03356249 (registered November 29, 2017) https://clinicaltrials.gov/ct2/show/NCT03356249

## Abstract 2

Supplemental Digital Content is available in the text

## 1 Background

### 1.1 Background and rationale

Sepsis remains a challenge in intensive care medicine, its incidence increasing continuously over the past decades.^[1–3]^ Despite massive efforts in sepsis research, new therapeutic approaches are rare and mortality in patients with septic shock still remains unacceptably high.^[1–3]^ In addition to an early focus control, recent guidelines recommend the initiation of an empiric antibiotic therapy as early as possible (preferably within 1 hour) following diagnosis of sepsis.^[4]^ However, the identification of the causative pathogen is crucial for early optimization of the antimicrobial treatment regime. In this context, culture-based diagnostic procedures (e.g., blood cultures) represent the standard of care, although they are associated with relevant limitations^[4,5]^: (i) depending on microbiological growth, it might take up to several days until final results (including pathogen identification and resistance patterns) are available, (ii) culture-based diagnostic procedures often reveal false negative results due to the administration of an empiric antibiotic therapy, and (iii) may however also reveal false positive results due to microbial contaminations (e.g., not strictly aseptic blood sample collection). Accordingly, patients suffering from sepsis or septic shock are at high risk for antimicrobial overtreatment, antibiotics-related toxicity, and the selection of multidrug-resistant pathogens due to an inadequate and prolonged use of broad-spectrum antibiotics. In this context, culture-independent molecular diagnostic procedures [e.g., polymerase chain reaction (PCR)-based techniques] have already been introduced for the identification of the causative pathogen in infected patients.^[6–10]^ However, the occurrence of ambiguous results as well as limitations in the quantitative measurement of the bacterial load in patients’ samples and detection of antibiotic resistance markers are known limitations of these PCR-based diagnostic approaches. Therefore, the concept of an unbiased sequence analyses of circulating cell-free deoxyribonucleic acid (cfDNA) from plasma samples of septic patients by next-generation sequencing (NGS) has recently been identified to be a promising diagnostic platform for critically ill patients suffering from bloodstream infections.^[11,12]^ This new NGS-based approach provides a basis to differentiate the relevant infecting organisms and to rule out potential microbial contaminants (e.g., coagulase-negative staphylococci) by establishing a quantitative score [sepsis indicating quantifier (SIQ) score]. This goes beyond state-of-the-art molecular approaches for the diagnosis of infecting organisms in septic specimens, which are not open but instead based on PCR amplification of defined targets and are, in most cases, just qualitative in nature. Therefore, this approach allows for an unbiased analysis of bloodstream infections, which might be especially useful for the diagnosis of cases where classic microbiological or molecular diagnostic approaches fail. However, although this new approach might be more sensitive and specific than state-of-the-art technologies, additional clinical trials are needed to exactly define the performance as well as the clinical value, as the presented studies were limited by the low number of patients.

### 1.2 Question and justification of the project (rationale)

The objective of this prospective, observational, noninterventional, multicenter study on the diagnostic use of NGS in patients suffering from sepsis is conducted to provide evidence of the effectiveness of a NGS-based approach in the quantitative measurement of the bacterial load in patients’ blood samples-a clinical trial on a new technique in an old diagnostic problem field.

### 1.3 Objectives

*Next GeneSiS* primarily evaluates the performance of a NGS-based approach for the detection of the relevant infecting organisms in patients with suspected or proven sepsis by the use of the quantitative sepsis indicating quantifier (SIQ) score in comparison to standard (culture-based) microbiological diagnostics. Secondarily, the clinical value of this approach will be estimated by a panel of independent clinical specialists, retrospectively identifying potential changes in patients’ management based on NGS results.

The following aspects have been defined as further secondary objectives:

(1) Evaluation of antimicrobial resistance patterns and virulence factors;
(2) Evaluation of process times for NGS-based measurements;
(3) Diagnostic or prognostic value of host nucleosome positioning patterns derived from plasma cell-free DNA in patients with suspected or proven sepsis;
(4) Diagnostic value of host expression profiles including RNA-derived biomarkers in patients with suspected or proven sepsis;
(5) Diagnostic or prognostic value of methylglyoxal (MG)-derived carbonyl stress in patients with suspected or proven sepsis.

### 1.4 Trial design

*Next GeneSiS* is a prospective, observational, noninterventional, multicenter study.

This study protocol follows the Standard Protocol Items: Recommendations for Interventional Trials (SPIRIT) guidelines (see Supplemental File 1).

## 2 Methods

### 2.1 Study setting

*Next GeneSiS* is conducted in terms of a multicenter study on medical as well as surgical intensive care units (ICUs) of maximum care hospitals throughout the *Translational Intensive Care Research Network on Organ Dysfunction* (TIFOnet) in Germany. Coordinating center of the study is the Department of Anesthesiology, Heidelberg University Hospital supported by the Coordination Centre for Clinical Trials (KKS) Heidelberg. Data management and statistical analysis are provided by the Institute for Medical Biometry and Informatics (IMBI), Heidelberg. Fraunhofer IGB provides next-generation sequencing (NGS) devices and is responsible for NGS-based measurements as well as calculation of the SIQ score in plasma samples of included septic patients.

### 2.2 Eligibility criteria

Patients with sepsis or septic shock according to the new sepsis definitions (Sepsis-3)^[13]^ with an onset < 24 hours are eligible for study inclusion. A summary of all inclusion and exclusion criteria for participants are provided in Table 1.

**Table 1:**
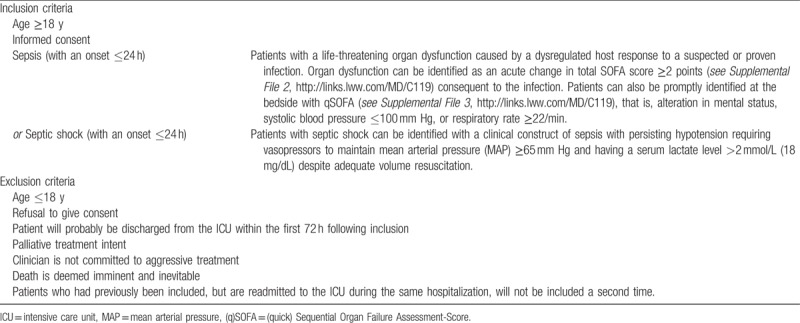
Inclusion and exclusion criteria of Next GeneSiS-trial.

### 2.3 Interventions

None.

### 2.4 Outcomes

For the evaluation of NGS-performance (sensitivity, specificity, positive predictive value, negative predictive value), results of the NGS-based approach for each sample will be compared with those obtained using conventional microbiology methods for the same sample. Moreover, interobserver agreement will be assessed by the calculation of Cohens Kappa.

The clinical value of the NGS-based approach will be estimated by a panel of 3 independent clinical specialists not associated with the study site, retrospectively identifying potential changes in patients’ management based on NGS results. Therefore, the panel will be provided with clinical case summaries, NGS results, and standard-of-care results from all samples tested. Results of microbiological routine diagnostics in specimens different from blood [e.g., body fluid, tissue, bronchoalveolar lavage fluid (BALF), endotracheal aspirate] will be included when they have been obtained within a timeframe of ≤72 hours prior or after the timepoints for NGS-based measurements. To identify potential changes in antimicrobial management that may have occurred if the results from the NGS technology had been available for clinical use, the panel will be provided with a special questionnaire.

Secondary subgroup analyses will focus on the clinical value especially for patients suffering from a failure of empiric treatment within the first 3 days after onset [as assessed by death of the patient or a lack of improvement of the patient's clinical condition (in terms of an inadequate decrease of SOFA-score) or persistent high procalcitonin levels].

### 2.5 Description of the used methods

#### 2.5.1 Standard-of-care microbiological analyses

Standard-of-care microbiological analyses of potential pathogens in the different specimens (also including rare pathogens of opportunistic infections in immunocompromised patients such as *Aspergillus* spp., *Mucorales*, *Pneumocystis jirovecii*, *Cryptococcus neoformans*, etc.) will be performed according to the usual practice in each participating institution.

Blood culture testing in Heidelberg University Hospital is routinely performed as described in.^[14]^ In brief, whole blood samples are obtained via direct venipuncture (e.g., antecubital vein) applying sterile techniques and 10 mL blood is inoculated to both an aerobic and an anaerobic liquid culture medium (BACTEC PLUS, BD Biosciences, Heidelberg, Germany). Cultures are incubated for at least 5 days (BACTEC, BD Biosciences, Heidelberg, Germany) and positive cultures are analyzed according to approved inhouse hospital standard techniques, including identification by VITEK2 (Biomerieux, Nuertingen, Germany) or MALDI TOF (Bruker, Madison, WI) and automated antimicrobial susceptibility testing (VITEK 2). Quantification of HSV1 DNA and cytomegalovirus DNA from plasma or tracheal secretion is performed via quantitative real time PCR as previously described.^[15]^ Cultivation of wound swabs, catheter, and stool samples is carried out as previously described.^[16,17]^

#### 2.5.2 Next-generation sequencing (NGS)

NGS-based measurements will be performed as described previously.^[11]^ In detail, plasma samples for NGS will be prepared from EDTA-anticoagulated blood tubes (Sarstedt S-Monovette 9 ml K3E) by centrifugation for 10 minutes at 2500*g* (according to manufacturer's instructions) and further storage in Eppendorf tubes (with a plasma volume of 1 ml in each tube) at −80°C until further processing. Plasma sample preparation (starting with the initial blood draw until storage of plasma samples at -80°C) should take no longer than 4 hours. Transfer of plasma samples to Fraunhofer IGB (Stuttgart, Germany) needs to be performed on dry ice. At Fraunhofer IGB, nucleic acids will be isolated from thawed plasma after a centrifugation step of 10 minutes at 16,000*g* with the QIAsymphony sample preparation (SP) instrument and the QIAsymphony DSP Circulating DNA Kit (Qiagen, Hilden, Germany) according to the manufacturer's protocol. Plasma volumes after centrifugation will be adjusted if necessary to 1200 μL with sterile phosphate buffered saline. Final elution of the nucleic acids from the spin column will be carried out with 60 μL molecular biology grade water (5 Prime, Hilden Germany). Contamination controls will be prepared following the same procedure, starting from 1200 μL molecular biology grade water (5 Prime, Hilden, Germany) and 1200 μL of sterile phosphate buffered saline, which will be prepared using the Circulating Nucleic Acid Kit (Qiagen, Hilden, Germany). The cfDNA will be quantified with the Qubit dsDNA HS Assay Kit (Life Technologies, Carlsbad, CA) and quality will be assessed with the HS NGS.

#### 2.5.3 Fragment analysis kit and the fragment analyzer instrument (AATI)

Libraries for NGS are prepared from 1 ng cfDNA using the Nextera XT library preparation Kit (Illumina, San Diego, CA) according to the manufacturer's protocol, with a Biomek FXP liquid handling robot (Beckman Coulter, Brea, CA). The final elution will be carried out in 35 μL of resuspension buffer (Illumina, San Diego, CA). A further contamination control is added by using 5 μL of molecular biology grade water (5 Prime, Hilden, Germany) as template for the Nextera XT Library Preparation Kit (Illumina, San Diego, CA). Sequencing of the libraries are performed on a HiSeq2500 (Illumina, San Diego, CA), resulting in 25 to 30 million 100-bp single end reads, on average, per sample. Raw reads are cleared from potential adapter contamination, quality controlled, and, if necessary, trimmed using BBDuk (https://sourceforge.net/projects/bbmap/). To pass the quality filter, read quality needs to surpass a Phred score of 20 and achieve a minimal length of 50 bp after trimming of low quality and adapter bases. Subsequently, NextGenMap is used to align quality-controlled reads to the human reference genome (hg19) requiring a minimum identity between read and reference genome of 80%. Reads mapping to the human reference genome and reads with low complexity (consecutive stretches of di- and trinucleotides along the whole read sequence) are excluded from further analysis.^[18]^ Finally, Kraken is used to assign reads to systematic classification using the RefSeq database (release version 68) comprising 35,749 bacterial and 4340 viral genomes complemented by 12 selected fungal genomes. As several Xanthomonas species are described as well-known contaminants, Xanthomonas reads are excluded as well as the Illumina sequencing spike-in PhiX.^[19]^ To quantitatively compare the number of reads that map to different microbial taxonomic classifications between different samples, the read counts are normalized by the respective library size.

#### 2.5.4 Sepsis Indicating Quantifier (SIQ)-score

Within the recently published work,^[11]^ we introduced the n × (s + 1) dimensional count matrix D, where n is the number of control samples and s the number of species detected in all samples. Thus, D_ij_ defines the number of reads found in control sample i for species j. D_i,(s__+__1)_ defines the number of reads which cannot be assigned to any species. One notes that D_i,(s + 1)_ is usually larger than the D_ijs_. Then, Eq. 1 is the maximum likelihood estimate of the probability to observe species j in a control sample:
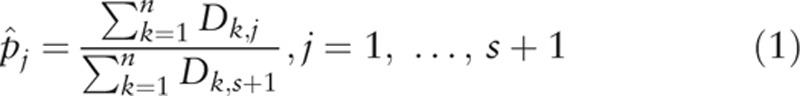


As the number of reads for 1 species is typically low, we assumed that the read counts for species j are Poisson distributed with parameter:
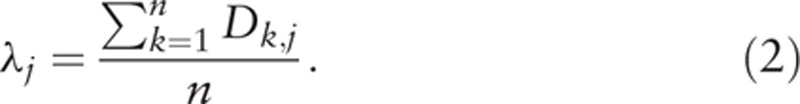


To test this assumption for each species, a standard χ^2^ goodness of fit test is performed. For reads sequenced from patient plasma, the same data processing pipeline is applied, which yields a read count vector C = (C_1_, …, C_s_, C_s + 1_). On the basis of the Poisson distribution with species-specific parameter λ_j_, the *P* value to observe at least C_j_ read counts in a patient sample is computed as:
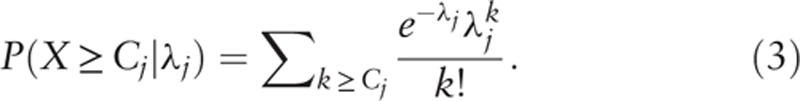


If this *P* value is small, then one would reject the hypothesis that the read count of species j in the patient sample follows the Poisson distribution derived from the healthy individuals and conclude that the respective species occurs too often in the patient. Now, with the given species specific λ, we can compute the SIQ score as follows:
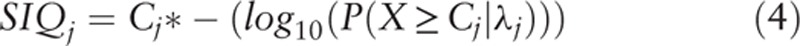


The SIQ score now gives rise to a quantitative and probabilistic assessment of every detected microbe in the respective sample.

### 2.6 Data collection

The following baseline data will be collected upon enrolment: patient demographics (e.g., age and sex), date and time of hospital and ICU admission, admission source (e.g., emergency department, outpatient clinic/referral, operating room, postanesthesia care unit, and other hospital unit), major comorbid conditions, immune status (host factors predisposing for an immunodeficiency according to^[20]^; *see Supplemental File 4*), site of suspected or confirmed infection, antimicrobial course before study enrolment, surgery/procedures for suspected site of infection before enrolment, and Sequential Organ Failure Assessment (SOFA) Score. Clinical data collection during admission will include pertinent laboratory data, use of mechanical ventilation, and antimicrobial/antibiotic therapy including duration of therapy, and date of therapy will be initiated and discontinued. Vasoactive therapy, renal replacement therapy, surgical and other procedures for diagnosis/treatment of infection, radiological testing for diagnosis/evaluation of potential infection, indwelling vascular access devices, and vital status will also be recorded. Discharge data will include date of discharge (ICU and hospital), discharge destination (general hospital floor, skilled nursing facility, and home), and vital status at discharge (survival/death).

### 2.7 Participant timeline

**Figure 1:**
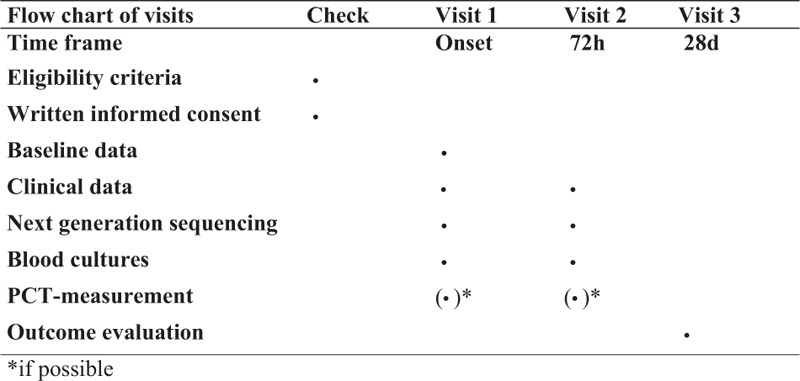
Detailed flow chart of specific procedures, assessments and visits (Spirit figure). PCT = procalcitonin.

Two sets of blood cultures (2x aerobic/2x anaerobic) will be collected at study inclusion (=Onset) as well as 72 hours afterwards (=72** **h). In parallel, plasma samples for NGS-based measurements need to be obtained as described previously. Further blood samples for NGS-based measurements can be collected whenever physicians order blood cultures (2x aerobic/2x anaerobic) because of the clinical suspicion of a blood stream infection (BSI) within the first 3 days after study inclusion. Results of microbiological routine diagnostics in specimens different from blood (e.g., body fluid, tissue, broncoalveolar lavage, endotracheal aspirate) will be used for further analyses when they are obtained within a timeframe of ≤72 hours prior or after the timepoints for NGS-based measurements. Clinical data collection and (if possible) PCT measurements will be performed at onset as well as at 72** **hours after study inclusion. The final outcome evaluation of patients will be performed at 28 days. A detailed flow chart of the trial specific procedures, assessments, and visits for participants is provided in Fig. 1.

### 2.8 Sample size

*Next GeneSiS* is performed in terms of an exploratory pilot study and is therefore not statistically powered. We estimate that approximately 500 patients need to be enrolled to enable a reasonable assessment of the performance of the NGS-based approach when compared with standard-of-care microbiology.

### 2.9 Recruitment

All adult patients (≥18 years) admitted to the participating centers for the management of suspected or proven sepsis will be considered for inclusion in this prospective study.

### 2.10 Data collection methods

All data collected in this trial will be recorded on standardized electronic case report forms (eCRF), which have to ensure full documentation of all patient data required by the study protocol. The investigators are responsible for ensuring that all parts of the eCRFs are filled in correctly.

### 2.11 Data management

All protocol-required information collected during the trial must be entered by the investigator, or designated representative, in the electronic case report form (eCRF). The investigator, or designated representative, should complete the eCRF pages as soon as possible after information is collected, preferably on the same day that a trial subject is seen for a trial procedure. Any outstanding entries must be completed as soon as possible. The completed eCRF must be approved by the investigator or by a designated subinvestigator.

The approved eCRF is then sent to IMBI being in charge of the data management within the trial. In order to ensure that the database reproduces the eCRFs correctly, the IMBI accomplishes a double entry of data to the statistical program SAS. IMBI representatives will check completeness, validity, and plausibility of data using validating programs, which will generate queries. All validation rules will be predefined in a data validation plan. The investigator or the designated representatives are obliged to clarify or explain the queries. If no further corrections are to be made in the database, it will be closed and used for statistical analysis. The data will be managed and analyzed according to the appropriate standard operation procedure (SOP) valid in the IMBI. According to §13 of the GCP Ordinance,^[21]^ all important trial documents (e.g., CRFs) are archived for at least 10 years after completion of the clinical trial.

### 2.12 Statistical methods

For the evaluation of NGS-performance, results obtained with the NGS technology for each sample will be compared with those obtained using conventional microbiology methods for the same sample. Agreement and concordance will be assessed using a McNemar test and Cohen κ. All percentages and confidence intervals (CIs) for proportions will be calculated using the exact method and are rounded to the nearest percentage. Direct comparison of positive and negative results will be conducted with organism identification for each method (conventional microbiology vs NGS). Coagulase-negative Staphylococcus, xanthomonas, and other common skin contaminants will be annotated as “potential contaminants” for both methods and will be excluded from the overall analysis. Discrepant results between the NGS and culture-based techniques cannot be directly confirmed by an independent method. Two approaches will be used to resolve such discrepancies. In a subset of patients, multiple samples will be collected per standard-of-care. This includes 2 independent fresh venipunctures (left arm vs right arm) or 1 venipuncture and 1 sample collected from an indwelling line. Paired analysis of NGS testing results between these independently collected samples will be conducted to indicate the likelihood of true infection. In addition, independent clinical adjudication (described below) will be performed using all the clinical data collected as part of the study, including standard-of-care microbiology results and NGS results.

The clinical value of the NGS-based approach will be estimated by a panel of 3 independent clinical specialists not associated with the study site by the use of a special questionnaire as described above. Analyses of the reviewers’ independent responses will be performed using a majority rule such that 2 of 3 responses for a given patient determine the outcome for that patient. Afterwards, special subgroup analyses (χ^2^ test for categorical data and further methods of variance analysis for continuous data) will focus on the clinical value especially for patients suffering from a failure of empiric treatment within the first 3 days after onset [as assessed by death of the patient or lack of improvement of the patient's clinical condition (in terms of an inadequate decrease of SOFA-score) or persistent high procalcitonin levels]. In addition, graphs are presented where possible. All statistical tests will be performed using SAS (SAS Institute, Cary, NC). A *P* value of less than .05 will be considered statistically significant.

### 2.13 Data monitoring

If necessary, monitoring will be done by personal visits from a clinical monitor of the Department of Anesthesiology, Heidelberg University Hospital. The monitor will review the entries into the eCRFs on the basis of source documents. The investigator must allow the monitor to verify all essential documents and must provide support at all times to the monitor. By frequent communications (letters, telephone, fax), the site monitor will ensure that the trial is conducted according to the protocol and regulatory requirements.

### 2.14 Harms

Due to the noninterventional character of *Next GeneSiS*, study-related adverse events (AEs) are restricted to complications of study-related blood draws (local lesions at puncture site or volume of blood draws). Such minor AEs are recorded in the eCRF. Serious AEs (SAEs) resulting in death, a life-threatening state, a prolongation of existing hospitalization, a persistent or significant disability or incapacity due to study participation are not expected.

### 2.15 Auditing

Regular audits by the sponsor are not intended. For the purpose of on-site inspection or audit, the competent authorities may require access to all source documents, CRF, and other trial-related records. The investigator must ensure availability of these documents and support the work at any time.

### 2.16 Ethics

Described procedures are meant to ensure that all parties involved abide by the principles of Good Clinical Practice (GCP)^[21,22]^ and those stipulated in the Declaration of Helsinki.^[23]^ The conducting takes place in accordance with local statutory and implementing provisions.

### 2.17 Research ethics approval

Before the beginning of the clinical trial, the study protocol, the patient information and informed consent, and all other required documents will be submitted to the competent ethical review committees of all participating centers. A first positive ethical vote has been given by the Ethics Committee of the Medical Faculty of Heidelberg, Trial Code No. S-084/2017.

### 2.18 Protocol amendments

Changes to the protocol are made in writing and require the approval of all signatories of the protocol. Subsequent amendments also require a positive assessment from the competent ethics committee.

### 2.19 Consent or assent

The members of the study group must inform eligible patients, both orally and in writing in an intelligible form about nature, significance, and implications. Before participants can be enrolled in *Next GeneSiS*, they must consent to participation in writing. For potential trial participants who are incapable, there is another procedure.

If a legal guardian already exists, they are duly informed in accordance with the regulations and subsequently consent to participation in writing. If no legal guardian exists, participants are enrolled in the clinical trial after a near family member has been informed about nature, significance, and implications and has agreed to participation in the study mindful of the interest of the patient concerned (also by telephone). In summary proceedings, the designation of a legal guardian is begun at the district court. If no near family member is available, participants are enrolled after a guardianship judge has been informed about nature, significance, and has agreed to participation in the study mindful of the interest of the patient concerned (also by telephone). A near family member is appointed legal guardian earliest possible; they are duly informed in accordance with the regulations and subsequently consent to participation in writing (delayed consent). In any case, informed consent of study participants is sought retrospectively once they are capable of giving consent again.

### 2.20 Confidentiality

Data collected are handled in accordance with the provisions of the Federal Data Protection Act (BDSG).^[24]^ During the clinical trial, participants are solely identified by a distinct reference number. For storage on a computer, the provisions of the BDSG^[24]^ are abided by. Data are handled with strict confidentiality. For protection of these data, organizational measures are taken to prevent disclosure to unauthorized third parties. The relevant rules of the country-specific data legislation are complied with.

## 3 Discussion

In patients suffering from sepsis or septic shock, positive blood cultures are obtained in only a fraction of cases despite proven underlying bacterial infection of 33%.^[25–27]^ This is partially attributable not only to technical shortfalls in blood culture acquisition but also due to local foci, fastidious organisms, or very low rates of viable microorganisms in blood stream.^[28]^ A molecular approach with higher sensitivity for sepsis might be accomplished by a potentially increased release of microbial cfDNA from acute inflammatory processes. In this context, NGS-based testing exhibits several advantages over PCR assays: the data-driven diagnosis without premonition of suspected species, no need for specific primer design, and the opportunity to detect bacterial, fungal, and viral pathogens in a single assay. Although NGS technology becomes increasingly important in clinical microbiology (e.g., for strain typing or microbiome studies), to date, only sporadic reports of NGS-analyzed clinical specimens have been published, including 1 actionable single-case report of NGS-based detection of Leptospira from cerebrospinal fluid.^[29–34]^ But so far, none of these reports aiming at NGS-based diagnosis of bacterial infections include microbial classification/calling strategies formulated by significance and quantitation values. Especially in specimens such as plasma, where only lowest amounts of microbial DNA can be expected, the sensitivity of NGS is challenged by the detection of bacterial contaminants of laboratory reagents and workflows.^[19,35]^ We therefore developed the SIQ score to discriminate relevant DNA fragments from noise caused by contaminant or commensal species, which has been used successfully in a small retrospective case series as well as in 1 prospective observational clinical cohort study of patients suffering from septic shock.^[11,12]^ A systematic reevaluation of these promising results in a larger cohort of patients suffering from sepsis is subject of the presented *Next GeneSiS*-trial.

### 3.1 Justification for enrolment of participants not capable of giving consent

Bloodstream infections remain one of the major challenges in ICUs, leading to sepsis or even septic shock in many cases. Due to the lack of timely diagnostic approaches with sufficient sensitivity, mortality rates of sepsis are still unacceptably high. However, a prompt diagnosis of the causative microorganism is critical to significantly improve outcome of bloodstream infections. Although various targeted molecular tests for blood samples are available, time-consuming blood culture-based approaches still represent the standard of care for the identification of bacteria. With regard to these alarming figures, the current clinical trial (*Next GeneSiS*) is designed to investigate a new diagnostic approach, namely, NGS. The majority of the patients affected are sedated and given artificial ventilation. Even before sedated, affected patients sometimes need to be regarded incapable of giving consent due to the underlying severe infection, inflammatory response, and severe pain. Therefore, in these cases, informed consent to participate in *Next GeneSiS* needs to be given by a legal guardian until the affected patient is capable of consent. Nevertheless, especially, these critically ill patients need to be enrolled in *Next GeneSiS,* in order to assess the diagnostic value of the above-described NGS-based approach for the early detection of the causative microorganism in sepsis. This might help to improve outcome of patients suffering from sepsis due to an early optimization of the anti-infective treatment regime. This might especially be true for patients, where classic microbiological or molecular diagnostic approaches fail.

## Acknowledgments

The authors are indebted to the Institute of Medical Biometry and Informatics, Heidelberg, and the Coordination Centre for Clinical Trials Heidelberg for ongoing support. We acknowledge financial support by Deutsche Forschungsgemeinschaft within the funding program Open Access Publishing, by the Baden-Württemberg Ministry of Science, Research and the Arts, and by Ruprecht-Karls-Universität Heidelberg.

## Supplementary Material

Supplemental Digital Content
